# Supplementary figures and images for: Lysozyme Amyloid Fibril Structural Variability Dependence on Initial Protein Folding State
Source: Int J Mol Sci. 2022 May 12;23(10):5421. doi: 10.3390/ijms23105421 (PMC9141980; doi:10.3390/ijms23105421)

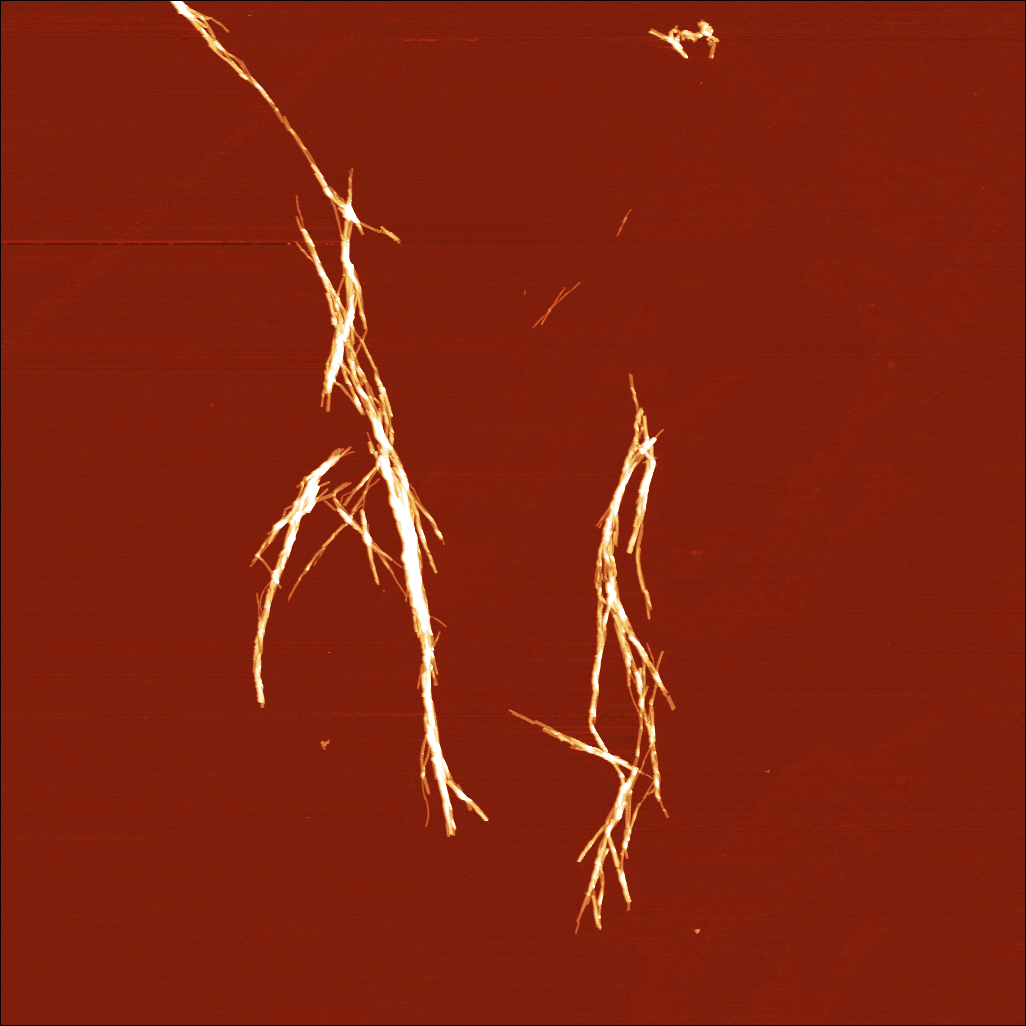

Supplement: Supplementary file 1 [file ijms-23-05421-s001.zip › Type 1 AFM.tiff]

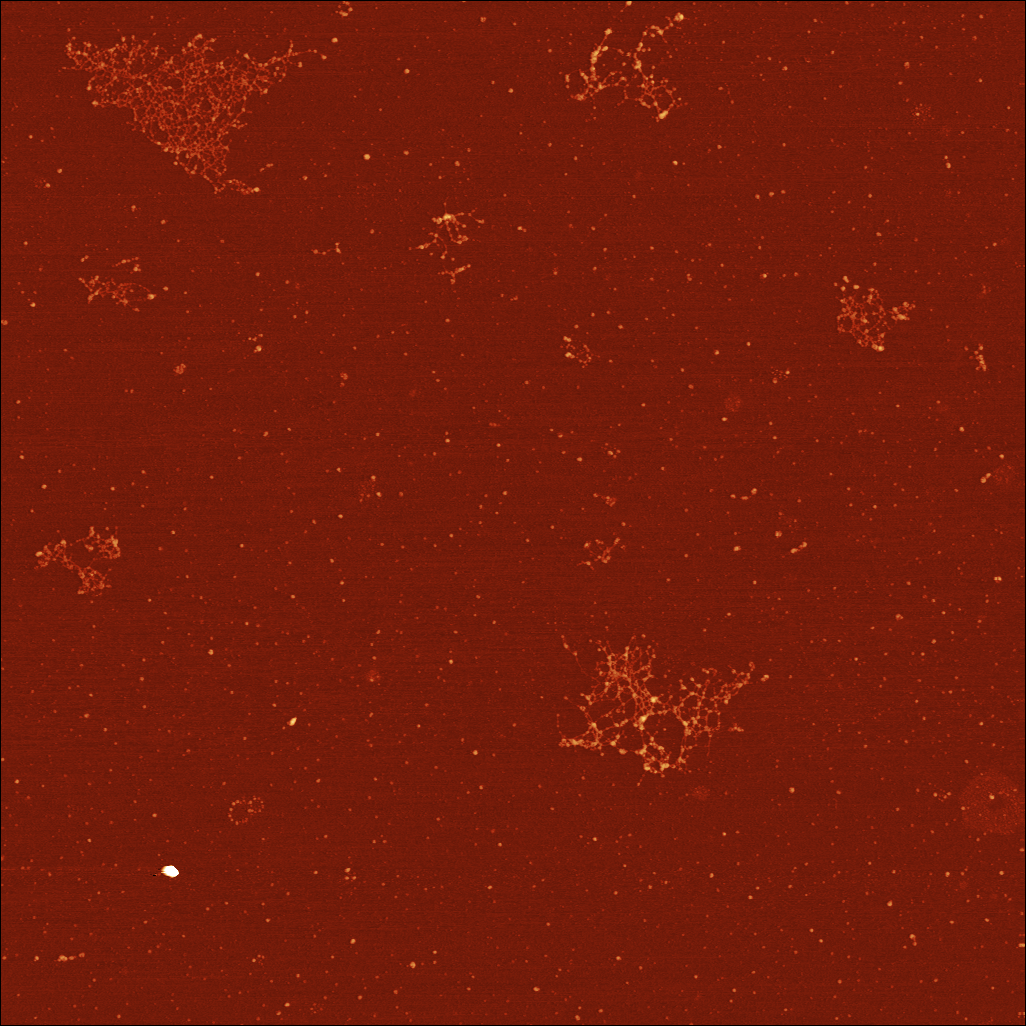

Supplement: Supplementary file 1 [file ijms-23-05421-s001.zip › Type 2 AFM.tiff]

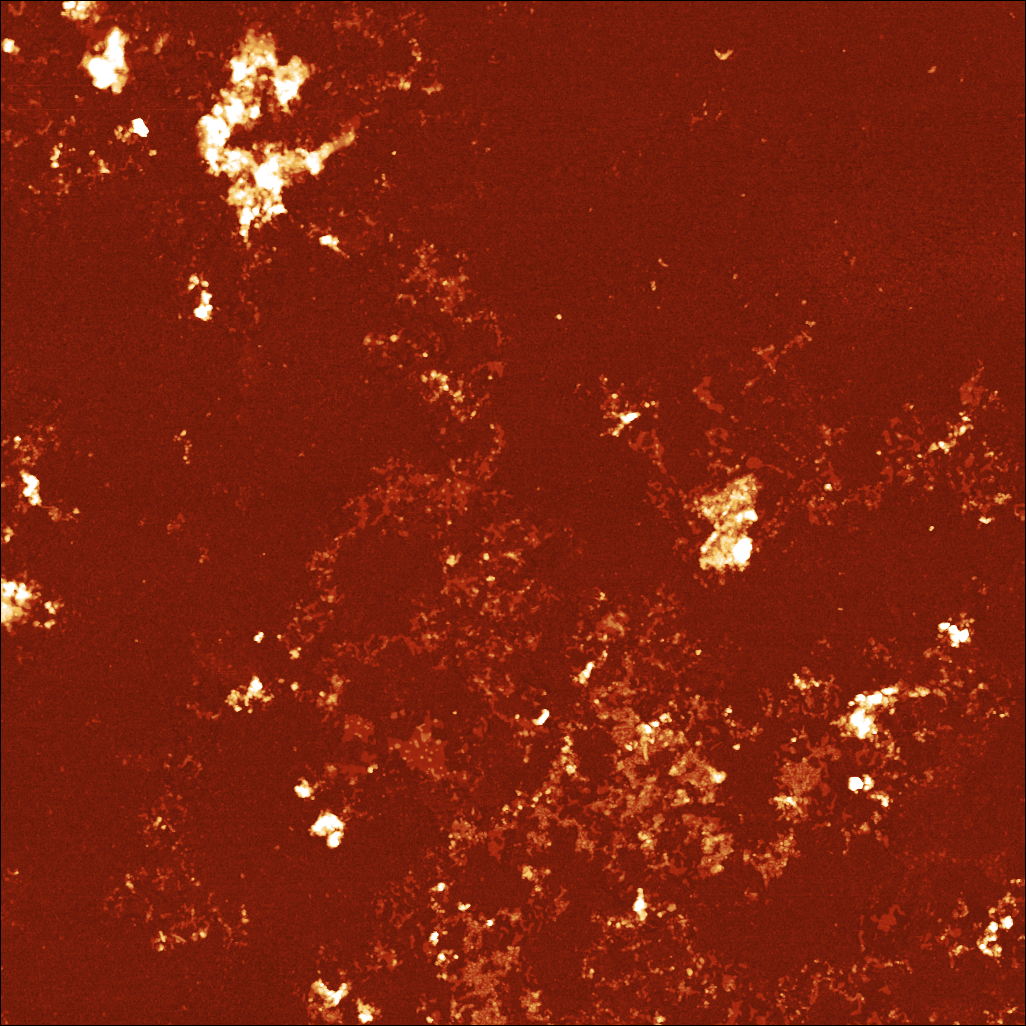

Supplement: Supplementary file 1 [file ijms-23-05421-s001.zip › Type 3 AFM.tiff]
